# Supplementary material for: A dose-dependent beneficial effect of methotrexate on the risk of interstitial lung disease in rheumatoid arthritis patients
Source: PLoS One. 2021 Apr 16;16(4):e0250339. doi: 10.1371/journal.pone.0250339 (PMC8051807; doi:10.1371/journal.pone.0250339)
Supplement: S1 File — (PDF) [file pone.0250339.s001.pdf]

Kwestionariusz pacjenta

Imię i nazwisko: …………………………………………………………

Data urodzenia: …………………………………………………………

Płeć: ……………………………………………………………………….

Wzrost: …………………………………………………………………….

Waga: ……………………………………………………………………….

Palenie papierosów: TAK NIE

Jeśli TAK, to: obecnie w przeszłości

liczba papierosów dziennie ………………..

od ……………………………………. do ……………………………

Data rozpoznania RZS: ………………………………………………..

Czy rozpoznano u Pana/ -i śródmiąższową chorobę płuc lub włóknienie płuc? TAK NIE

Jeśli TAK, to kiedy ………………………………………………………………………..

Czy rozpoznano u Pana/-i zatorowość płucną? TAK NIE

Jeśli Tak, to kiedy …………………………………………………………………………..

Czy rozpoznano u Pana/-i niewydolność serca? TAK NIE

Jeśli TAK, to kiedy ……………………………………………………………………………..

Inne choroby współistniejące:

………

………

………

………

………

Czy obecnie występują u Pana/-i objawy infekcji układu oddechowego (gorączka, kaszel, duszność, krwioplucie, bóle w klatce piersiowej)? TAK NIE

Jeśli TAK, to jakie objawy i od kiedy ………………………………………………………………………………

………

………

Dotychczasowe leczenie RZS (daty stosowania, dawka):

1. Methotrexat:

Czy obecnie przyjmuje Pan/-i metotreksat?: TAK NIE

Jeśli TAK, to:

od kiedy: ………………………………………………………………………………………

jaka dawka: ……………………………………………………………………………………

Czy w przeszłości przyjmował/-a Pan/-i metotreksat? TAK NIE

Jeśli TAK, to:

Od kiedy do kiedy w jakich dawkach
……….

………..

……

……

2. Leflunomid

Czy obecnie przyjmuje Pan/-i leflunomid?: TAK NIE

Jeśli TAK, to:

od kiedy: ………………………………………………………………………………………

jaka dawka: ……………………………………………………………………………………

Czy w przeszłości przyjmował/-a Pan/-i leflunomid? TAK NIE

Jeśli TAK, to:

Od kiedy do kiedy w jakich dawkach
……….

………..

……

……

3. Sulfasalazyna

Czy obecnie przyjmuje Pan/-i sulfasalazynę? TAK NIE

Jeśli TAK, to:

od kiedy: ……………………………………………………………………………………………

jaka dawka: …………………………………………………………………………………………

Czy w przeszłości przyjmował/-a Pan/-i sulfasalazynę? TAK NIE

Jeśli TAK, to:

Od kiedy do kiedy w jakich dawkach
……….

………..

……

……

4. Chlorochina

Czy obecnie przyjmuje Pan/-i chlorochinę? TAK NIE

Jeśli TAK, to:

od kiedy: ……………………………………………………………………………………………

jaka dawka: …………………………………………………………………………………………

Czy w przeszłości przyjmował/-a Pan/-i chlorochinę? TAK NIE

Jeśli TAK, to:

Od kiedy do kiedy w jakich dawkach
……….

………..

……

……

5. Cyklosporyna A

Czy obecnie przyjmuje Pan/-i cyklosporynę? TAK NIE

Jeśli TAK, to:

od kiedy: ……………………………………………………………………………………………

jaka dawka: …………………………………………………………………………………………

Czy w przeszłości przyjmował/-a Pan/-i cyklosporynę? TAK NIE

Jeśli TAK, to:

Od kiedy do kiedy w jakich dawkach
…….

……..

………

………

6. Sole złota

Czy obecnie przyjmuje Pan/-i sole złota? TAK NIE

Jeśli TAK, to:

od kiedy: ……………………………………………………………………………………………

jaka dawka: …………………………………………………………………………………………

Czy w przeszłości przyjmował/-a Pan/-i sole złota? TAK NIE

Jeśli TAK, to:

Od kiedy do kiedy w jakich dawkach
…….

……..

………

………

7. Etanercept

Czy obecnie przyjmuje Pan/-i etanercept? TAK NIE

Jeśli TAK, to:

od kiedy: ……………………………………………………………………………………………

jaka dawka: …………………………………………………………………………………………

Czy w przeszłości przyjmował/-a Pan/-i etanercept? TAK NIE

Jeśli TAK, to:

Od kiedy do kiedy w jakich dawkach
…….

……..

………

……….

8. Adalimumab

Czy obecnie przyjmuje Pan/-i adalimumab? TAK NIE

Jeśli TAK, to:

od kiedy: ………………………………………………………………………………………………

jaka dawka: ……………………………………………………………………………………….…

Czy w przeszłości przyjmował/-a Pan/-i adalimumab? TAK NIE

Jeśli TAK, to:

Od kiedy do kiedy w jakich dawkach
……….

………..

…….

…….

9. infliksymab

Czy obecnie przyjmuje Pan/-i infliksymab? TAK NIE

Jeśli TAK, to:

od kiedy: ………………………………………………………………………………………………

jaka dawka: ……………………………………………………………………………………….…

Czy w przeszłości przyjmował/-a Pan/-i infliksymab? TAK NIE

Jeśli TAK, to:

Od kiedy do kiedy w jakich dawkach
……….

………..

…….

…….

10. Rytkuksymab

Czy obecnie przyjmuje Pan/-i rytuksymab? TAK NIE

Jeśli TAK, to:

od kiedy: ………………………………………………………………………………………

jaka dawka: ……………………………………………………………………………………

Czy w przeszłości przyjmował/-a Pan/-i rytuksymab? TAK NIE

Jeśli TAK, to:

Od kiedy do kiedy w jakich dawkach
…….

……..

………

………

11. Tocilizumab

Czy obecnie przyjmuje Pan/-i tocilizumab? TAK NIE

Jeśli TAK, to:

od kiedy: ………………………………………………………………………………………

jaka dawka: ……………………………………………………………………………………

Czy w przeszłości przyjmował/-a Pan/-i tocilizumab? TAK NIE

Jeśli TAK, to:

Od kiedy do kiedy w jakich dawkach
…….

……..

………

………

12. Czy obecnie przyjmuje Pan/-i inne leki biologiczne? Tak NIE

Jeśli TAK, to:

jakie …………………………………………………………………………………………

od kiedy: ………………………………………………………………………………………

jaka dawka: ……………………………………………………………………………………

Czy w przeszłości przyjmował/-a Pan/-i inne leki biologiczne? TAK NIE

Jeśli TAK, to:

Jakie, od kiedy do kiedy w jakich dawkach

…….

……..

………

………

13. Glikokortykosteroidy (Encorton, Metypred)

Czy obecnie przyjmuje Pan/-i glikokortykosteroidy? TAK NIE

Jeśli TAK, to:

od kiedy: ………………………………………………………………………………………

jaka dawka: ……………………………………………………………………………………

Czy w przeszłości przyjmował/-a Pan/-i glikokortykosteroidy? TAK NIE

Jeśli TAK, to:

Od kiedy do kiedy w jakich dawkach

…….

……..

………

………
