# Supplementary material for: A dose-dependent beneficial effect of methotrexate on the risk of interstitial lung disease in rheumatoid arthritis patients
Source: PLoS One. 2021 Apr 16;16(4):e0250339. doi: 10.1371/journal.pone.0250339 (PMC8051807; doi:10.1371/journal.pone.0250339)
Supplement: S2 File — (PDF) [file pone.0250339.s002.pdf]

**Questionnaire**

Full name: ……………………………………………………….

Date of birth: ……………………………………………………….

Sex: …………………………………………………………………………

Height: ………………………………………………………………….

Weight: ………………………………………………………………….

Smoking: YES NO

If YES: current in the past

number of cigarettes daily ………………..

from ……………………………….. to ……………………………

Date of rheumatoid arthritis diagnosis: ……………………………………………………..

Diagnosis of interstitial lung disease/ lung fibrosis? YES NO

If YES, date of diagnosis …………………………………………………………..

Diagnosis of thromboembolism? YES NO

If YES, date of diagnosis ……………………………………………………………

Diagnosis of heart failure? YES NO

If YES, date of diagnosis ………………………………………………………………

Other comorbidities:

…………………………………………………………………………………………………………………………………………
…………………………………………………………………………………………………………………………………………
…………………………………………………………………………………………………………………………………………
…………………………………………………………………………………………………………………………………………
………………………………………………………………………………………………………………………………………..

Current symptoms of respiratory tract disease (fever, cough, dyspnoea, hemoptysis, chest pain):
YES NO

If YES, which symptoms and start date of symptoms ……………………………………………………..

…………………………………………………………………………………………………………………………………………
…………………………………………………………………………………………………………………………………………

Treatment of RA (dates, doses):

1. Methotrexate:

Current treatment with methotrexate? YES NO

If YES:

From (date): …………………………………………………………………………………………

Current dose: ……………………………………………………………………………………

Treatment with methotrexate in the past? YES NO

If YES:

dates and doses:
……………………………………………………………………………………………………………….

………………………………………………………………………………………………………………..

…………………………………………………………………………………………………………………

…………………………………………………………………………………………………………………

2. Leflunomide

Current treatment with leflunomide? YES NO

If YES:

From (date): …………………………………………………………………………………………

Current dose: ……………………………………………………………………………………

Treatment with leflunomide in the past? YES NO

If YES:

dates and doses:
……………………………………………………………………………………………………………….

………………………………………………………………………………………………………………..

…………………………………………………………………………………………………………………

…………………………………………………………………………………………………………………

3. Sulfasalazine

Current treatment with sulfasalazine? YES NO

If YES:

From (date): …………………………………………………………………………………………

Current dose: ………………………………………………………………………………………

Treatment with sulfasalazine in the past? YES NO

If YES:

dates and doses:
…………………………………………………………………………………………………………………

…………………………………………………………………………………………………………………

…………………………………………………………………………………………………………………

…………………………………………………………………………………………………………………

4. Chloroquine

Current treatment with chloroquine? YES NO

If YES:

From (date): …………………………………………………………………………………………

Current dose: ………………………………………………………………………………………

Treatment with chloroquine in the past? YES NO

If YES:

dates and doses:
…………………………………………………………………………………………………………………

…………………………………………………………………………………………………………………

…………………………………………………………………………………………………………………

…………………………………………………………………………………………………………………

5. Cyclosporine

Current treatment with cyclosporine? YES NO

If YES:

From (date): …………………………………………………………………………………………

Current dose: ………………………………………………………………………………………

Treatment with cyclosporine in the past? YES NO

If YES:

dates and doses:
……………………………………………………………………………………………………………………….

………………………………………………………………………………………………………………………..

…………………………………………………………………………………………………………………………

…………………………………………………………………………………………………………………………

6. Gold salts

Current treatment with gold salts? YES NO

If YES:

From (date): ………………………………………………………………………………………

Current dose: …………………………………………………………………………………

Treatment with gold salts in the past? YES NO

If YES:

dates and doses:
……………………………………………………………………………………………………………………….

………………………………………………………………………………………………………………………..

…………………………………………………………………………………………………………………………

…………………………………………………………………………………………………………………………

7. Etanercept

Current treatment with etanercept? YES NO

If YES:

From (date): ………………………………………………………………………………………

Current dose: …………………………………………………………………………………

Treatment with etanercept in the past? YES NO

If YES:

dates and doses:
……………………………………………………………………………………………………………………….

………………………………………………………………………………………………………………………..

…………………………………………………………………………………………………………………………

…………………………………………………………………………………………………………………………….

8. Adalimumab

Current treatment with adalimumab? YES NO

If YES:

From (date): ……………………………………………………………………………………………

Current dose: …………………………………………………………………………………………

Treatment with adalimumab in the past? YES NO

If YES:

dates and doses:
……………………………………………………………………………………………………………………….

………………………………………………………………………………………………………………………..

…………………………………………………………………………………………………………………………….

…………………………………………………………………………………………………………………………….

9. Infliximab

Current treatment with infliximab? YES NO

If YES:

From (date): ……………………………………………………………………………………………

Current dose: …………………………………………………………………………………………

Treatment with infliximab in the past? YES NO

If YES:

dates and doses:
……………………………………………………………………………………………………………………….

………………………………………………………………………………………………………………………..

…………………………………………………………………………………………………………………………….

…………………………………………………………………………………………………………………………….

10. Rituximab

Current treatment with rituximab? YES NO

If YES:

From (date): ……………………………………………………………………………………………

Current dose: ………………………………………………………………………………………

Treatment with rituximab in the past? YES NO

If YES:

dates and doses: ……………………………………………………………………………………………………………….

………………………………………………………………………………………………………………………..

…………………………………………………………………………………………………………………………

…………………………………………………………………………………………………………………………

11. Tocilizumab

Current treatment with tocilizumab? YES NO

If YES:

From (date): ……………………………………………………………………………………………

Current dose: ………………………………………………………………………………………

Treatment with tocilizumab in the past? YES NO

If YES:

dates and doses: ……………………………………………………………………………………………………………….

………………………………………………………………………………………………………………………..

…………………………………………………………………………………………………………………………

…………………………………………………………………………………………………………………………

12. Current treatment with other biologics? YES NO

If YES:

which biologic ………………………………………………………………………………………………

from (date): ……………………………………………………………………………………………

current dose: ………………………………………………………………………………………

Treatmeny with other biologics in the past? YES NO

If YES:

which, dates and doses
……………………………………………………………………………………………………………………….

………………………………………………………………………………………………………………………..

…………………………………………………………………………………………………………………………….

…………………………………………………………………………………………………………………………….

13. Glucocorticosteroids (prednison,methyloprednisolon)

Current treatment with glucocorticosteroids? YES NO

If YES:

From (date): …………………………………………………………………………………………

Current dose: ……………………………………………………………………………………

Previous treatment with glucocorticosteroids? YES NO

If YES:

dates and doses:
……………………………………………………………………………………………………………………….

………………………………………………………………………………………………………………………..

…………………………………………………………………………………………………………………………….

…………………………………………………………………………………………………………………………….
